# Integrated microRNA and transcriptome profiling reveal key miRNA-mRNA interaction pairs associated with seed development in Tartary buckwheat (*Fagopyrum tataricum*)

**DOI:** 10.1186/s12870-021-02914-w

**Published:** 2021-03-09

**Authors:** Hongyou Li, Hengling Meng, Xiaoqian Sun, Jiao Deng, Taoxiong Shi, Liwei Zhu, Qiuyu Lv, Qingfu Chen

**Affiliations:** 1 Research Center of Buckwheat Industry Technology, Guizhou Normal University, Guiyang, 550001 China; 2 Key Laboratory of High-Quality Crops Cultivation and Safety Control of Yunnan Province, Honghe University, Honghe, 661100 China; 3 School of Life Sciences, Guizhou Normal University, Guiyang, 550025 China; 4 School of Big Data and Computer Science, Guizhou Normal University, Guiyang, 550025 China

**Keywords:** Tartary buckwheat, Integrated analysis, miRNAs, Target gene, Seed development, Seed size, Flavonoids, *DCL*, *AGO*, *RDR*

## Abstract

**Background:**

Tartary buckwheat seed development is an extremely complex process involving many gene regulatory pathways. MicroRNAs (miRNAs) have been identified as the important negative regulators of gene expression and performed crucial regulatory roles in various plant biological processes. However, whether miRNAs participate in Tartary buckwheat seed development remains unexplored.

**Results:**

In this study, we first identified 26 miRNA biosynthesis genes in the Tartary buckwheat genome and described their phylogeny and expression profiling. Then we performed small RNA (sRNA) sequencing for Tartary buckwheat seeds at three developmental stages to identify the miRNAs associated with seed development. In total, 230 miRNAs, including 101 conserved and 129 novel miRNAs, were first identified in Tartary buckwheat, and 3268 target genes were successfully predicted. Among these miRNAs, 76 exhibited differential expression during seed development, and 1534 target genes which correspond to 74 differentially expressed miRNAs (DEMs) were identified. Based on integrated analysis of DEMs and their targets expression, 65 miRNA-mRNA interaction pairs (25 DEMs corresponding to 65 target genes) were identified that exhibited significantly opposite expression during Tartary buckwheat seed development, and 6 of the miRNA-mRNA pairs were further verified by quantitative real-time polymerase chain reaction (qRT-PCR) and ligase-mediated rapid amplification of 5′ cDNA ends (5′-RLM-RACE). Functional annotation of the 65 target mRNAs showed that 56 miRNA-mRNA interaction pairs major involved in cell differentiation and proliferation, cell elongation, hormones response, organogenesis, embryo and endosperm development, seed size, mineral elements transport, and flavonoid biosynthesis, which indicated that they are the key miRNA-mRNA pairs for Tartary buckwheat seed development.

**Conclusions:**

Our findings provided insights for the first time into miRNA-mediated regulatory pathways in Tartary buckwheat seed development and suggested that miRNAs play important role in Tartary buckwheat seed development. These findings will be help to study the roles and regulatory mechanism of miRNAs in Tartary buckwheat seed development.

**Supplementary Information:**

The online version contains supplementary material available at 10.1186/s12870-021-02914-w.

## Background

MicroRNAs (miRNAs), a class of endogenous non-coding small RNAs (sRNAs) that are 20–24 nt in length, play crucial regulatory functions in animals and plants by repressing their target genes expression at the transcription or post-transcription level [1–4]. In plants, the biosynthesis of miRNAs is a multistep pathway that is involved in many genes and enzymes [5]. Plant miRNA genes are initially transcribed into primary miRNAs (pri-miRNAs) by RNA polymerase II in the nucleus [5, 6]. Then, the pri-miRNAs are processed into miRNA precursors (pre-miRNAs) with stem-loop structures by the DICER-LIKE1 (DCL1), HYPONASTIC LEAVES1 (HYL1) and SERRATE (SE) protein complex [5–7]. Next, these pri-miRNAs are further cleaved into miRNA::miRNA^*^ duplexes under the action of DCL1, HYL1 and SE protein complex, and then the 3΄ end of the duplexes are methylated by the methyltransferase HUA ENHANCER1 (HEN1) [5]. After methylation, the duplexes are exported to the cytoplasm by the HASTY protein [5, 8]. Finally, the miRNA duplex is bound by ARGONAUTE1 (AGO1) to form the RNA-induced silencing complex (RISC) to carry out its function by either cleaving target mRNAs or repressing the translation process [5, 9]. At present, miRNAs have been identified in numerous plants, and more and more evidence indicates that they play crucial roles in plant growth and development, secondary metabolism, biotic and abiotic stress tolerance, and signal transduction [4, 10–16].

Seed is the reproductive and the primary nutrient storage organ in many crop plants, and its developmental success or failure directly determines the final crop yield and seed quality, as well as whether genetic information can be successfully transmitted to the next generation [4]. Crop seed development is terribly a complex biological process that involves many gene regulatory pathways [17, 18]. An increasing body of evidence shows that miRNAs participate in the regulation of seed development in crop plants. To date, thousands miRNAs have been identified in the development of seed in multiple crop plants including rice [19, 20], maize [21, 22], wheat [23, 24], barley [25, 26], soybean [27, 28], peanut [18], *Brassica napus* [29, 30], narrow-leafed lupin [6], and common buckwheat [4] by using a high-throughput sequencing approach. These studies found that the expression of various miRNAs is extremely dynamic during seed development and some miRNAs are specifically expressed in seed, implying that miRNAs have very vital regulatory roles in seed development. In fact, a few miRNAs have been functionally demonstrated to play crucial regulatory roles in the seed development of model crop rice and a few other crops by negatively regulating their target genes expression. For example, miR159, miR160, miR397, miR398, and miR408 positively regulate rice grain size [31–35], while miR1432, miR156, miR167, miR396c, miR396e, miR396f, and miR530 have opposite roles in regulating the grain size [35–41]. Notably, the conserved role of miR408 and miR160 in regulating seed size was also found in *Arabidopsis* and tobacco, and cotton, respectively [42, 43]. In addition to the role in seed size, miRNAs were also demonstrated to regulate nutrient accumulation in developing seed [4]. For instance, miR160 positively regulate starch accumulation in rice seed [4, 32]. EgmiR5179 and csa-miR167A regulate the oil and linolenic acid biosynthesis in oil palm and *Camelina sativa* seeds, respectively [44, 45].

Tartary buckwheat (*Fagopyrum tataricum*) is an annual medicinal and edible crop, belonging to the eudicot family Polygonaceae [46]. It is widely cultivated in Asia and Eastern Europe, especially in the mountainous areas of Southwest China [46]. Tartary buckwheat seed is a good source of nutrients including starch, protein, dietary fiber, fatty acid (linoleic acid), and various minerals [46]. Importantly, Tartary buckwheat seed also contains rich flavonoids especially rutin, which have been proved to be effective in preventing liver injury and especially inflammatory liver injury [43, 46]. Therefore, it is of great significance to understand the molecular mechanism of Tartary buckwheat seed development, which will be helpful in high-yield and quality breeding of Tartary buckwheat [46]. To date, several transcriptome analyses have been reported that examine the molecular mechanism of Tartary buckwheat seed development [4, 43, 46–48]. However, to our knowledge, no one has studied miRNAs in Tartary buckwheat, and miRNAs whether and how to regulate the development of Tartary buckwheat seed is largely unclear. In this study, we first examined the conserved evolution of miRNA biosynthesis in Tartary buckwheat compared to other plants through homology identification and phylogenetic analysis of miRNA biosynthesis genes. Then, we identified the known and novel miRNAs in the developing seed of Tartary buckwheat and predicted their target genes. Finally, we performed integration analysis between miRNA and mRNA expression to insight into the miRNA-mediated molecular mechanisms of Tartary buckwheat seed development and identified key miRNA-mRNA interaction pairs for Tartary buckwheat seed development. Our results provide valuable information for enhancing the understanding of the miRNA-mediated regulatory mechanism of Tartary buckwheat seed development and aid in the Tartary buckwheat seed improvement.

## Results

### Identification, phylogeny, and expression profiles analysis of the miRNA biosynthesis genes in Tartary buckwheat

**Fig. 1:**
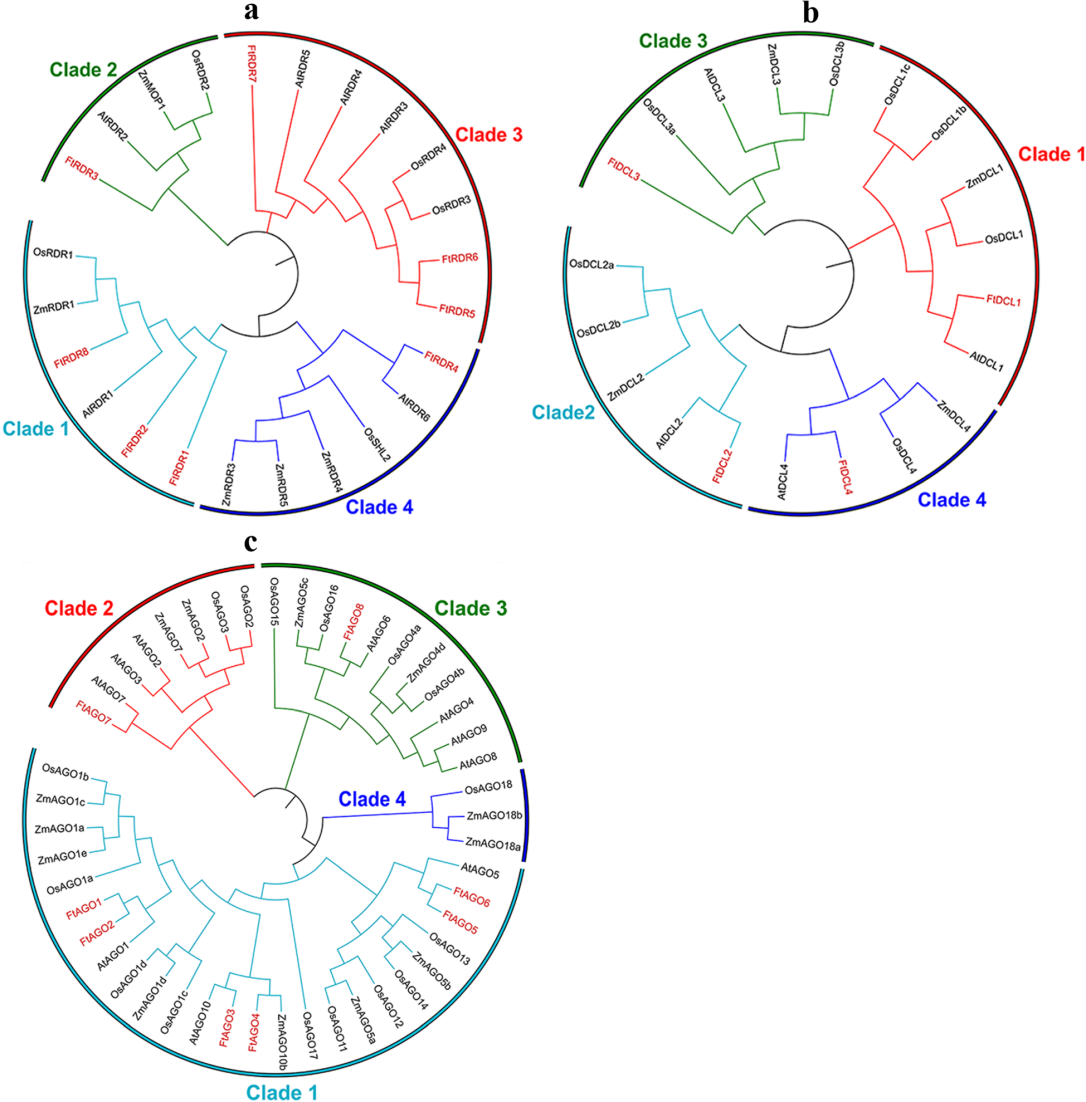
Phylogenetic analysis of Tartary buckwheat RDR, DCL and AGO proteins. The corresponding proteins of *Arabidopsis*, maize, and rice were also used to construct the phylogenetic tree. The phylogenetic tree was constructed by using MUSCLE and MEGA 7.0 with the Maximum Likelihood method

*RDR*, *DCL*, *HYL*, *SE*, *HEN*, *HST* and *AGO* genes have been demonstrated to play essential roles in plant miRNA biosynthesis [5]. As the first and most important step in studying the miRNA in Tartary buckwheat, we identified the orthologs of these genes in Tartary buckwheat. A total of 8 *RDR*, 4 *DCL*, 1 *HYL*, 2 *SE*, 1 *HEN*, 2 *HST*, and 8 *AGO* genes were identified in the Tartary buckwheat genome (Additional file 1: Table S1), respectively. Phylogenetic analysis revealed that plant RDR proteins can be divided into four clades, as previously defined by Qian et al. [49] (Fig. 1). Among the 8 FtRDR proteins, 3, 1, 3, and 1 belong to clades 1, 2, 3, and 4, respectively. In addition, the FtRDR proteins were closely homologous to *A. thaliana* RDR proteins. The DCL proteins can also be classed into four groups, and 4 FtDCL proteins showed a one-to-one counterpart with *A. thaliana* DCL proteins (Fig. 1). Similarly, the AGO proteins can be separated into four clades, but clade 4 only contained the grass AGO proteins as defined by Zhang et al. [50] (Fig. 1). For Tartary buckwheat AGO proteins, clade 1 was the biggest clade, which contained 6 FtAGO proteins. In contrast, both clades 1 and 2 only contained 1 FtAGO protein (Fig.1).

**Fig. 2:**
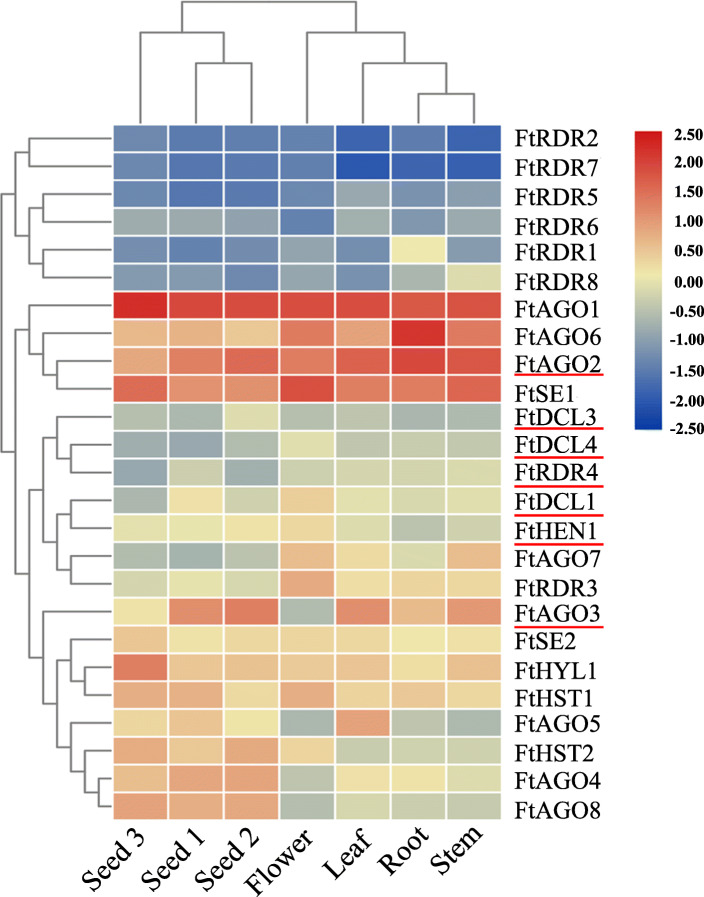
Expression profiles of Tartary buckwheat miRNA biosynthesis genes in different organs and during seed development. The different colors represent the expression abundance of the genes

RNA-seq data were further used to investigate the expression profiles of the 26 identified genes in roots, stems, leaves, flowers, and during seed development. As shown in Fig. 2, most *RDR* genes (6) had lower expression in all tissues. In contrast, 4 genes (*FtAGO1*, *FtAGO2*, *FtAGO6*, and *FtSE1*) had constitutive high expression in all tissues. The remaining 16 genes were expressed at a moderate level. Among them, 4 genes (*FtHYL1*, *FtHST2*, *FtAGO4*, and *FtAGO8*) exhibited specifically high expression in seeds. Furthermore, 6 genes (*FtDCL1*, *FtDCL3*, *FtDCL4*, *FtAGO2*, *FtAGO3,* and *FtRDR4*) showed significant differential expression (|log2(fold change)| > 1 and FDR value < 0.05) during seed development (Fig. 2). The 6 differentially expressed genes (DEGs) showed three expression patterns in the developing Tartary buckwheat seed (1) *FtAGO2* and *FtAGO3* were down-regulated only at the initial maturity stage (S3), (2) *FtDCL1* and *FtRDR4* showed a sustained decrease during seed development, and (3) *FtDCL3* and *FtDCL4* were up-regulated at the peak filling stage (S2) and down-regulated at the initial maturity stage (S3) (Fig. 2).

### Sequencing of Tartary buckwheat seed sRNAs

**Fig. 3:**
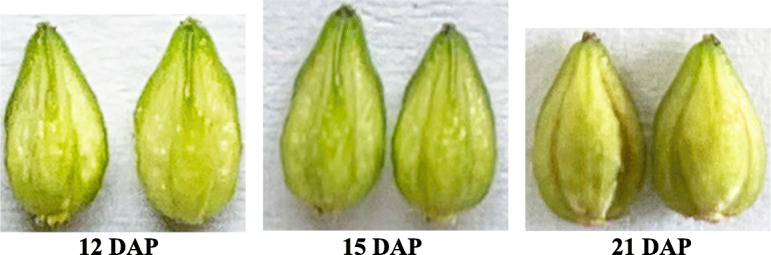
Seed phenotypes of Tartary buckwheat at the initial filling stage (S1), peak filling stage (S2), and initial maturity stage (S3)

To investigate the effects of miRNAs on seed development in Tartary buckwheat, we constructed and sequenced six sRNA libraries from seeds at three different developmental stages (Fig. 3). A total of 120.63 million raw reads were generated in six sRNA libraries (Additional file 1: Table S2). After filtration, 13.89 million, 17.09 million, 17.19 million, 14.54 million, 13.64 million, and 12.00 million clean reads were obtained for the six libraries, respectively (Additional file 1: Table S2). The length distribution displayed most the small RNA reads were 21–24 nt (Additional file 2: Figure S1). Among them, the 24 nt small RNA was the most abundant type and showed differential accumulation during Tartary buckwheat seed development (Additional file 2: Figure S1). To further obtain sRNA reads containing miRNAs, non-coding RNAs (including rRNAs, tRNAs, snRNAs, and snoRNAs) and repeat sequences were removed by comparing the clean reads to the Silv, GtRNAdb, Rfam, and Repbase databases. After discarding the non-coding RNAs and repeat sequences, a total of 10.45 million, 14.16 million, 14.60 million, 13.05 million, 6.80 million, and 5.63 million unannotated sRNA reads containing miRNAs were obtained for the six libraries, respectively (Additional file 1: Table S2). Among these unannotated clean reads, a total of 61.94, 66.76, 46.98, 39.20, 37.22, and 39.42% reads were mapped to the Tartary buckwheat reference genome, respectively (Additional file 1: Table S2).

### Identification of known and novel miRNAs

To identify known miRNAs in the developing Tartary buckwheat seed, the mapped sRNA reads were subjected to Blastn search against miRBase v21.0. In total, 101 conserved miRNAs, belonging to 25 known miRNA families, were identified (Additional file 1: Table S3). Of these families, 17 families contained more than two members, three families (MiR390, MiR399, and MiR858) contained two members, and the remaining five families (MiR159, MiR394, MiR397, MiR828, and MiR845) only contained one member. The length of these known miRNAs ranged from 19 to 22 nt, and the numbers of miRNAs with length of 19, 20, 21, and 22 nt were 8, 29, 56, and 8, respectively (Additional file 1: Table S3). Among these identified conserved miRNAs, 29 miRNAs were highly expressed, which have more than 1000 read counts at least in one library (Additional file 1: Table S3). The rest of miRNAs were expressed at a moderate level (36) or low level (36) which have < 10 read counts for each library, respectively (Additional file 1: Table S3). Notably, the highest expression miRNA was fta-miR159, and read counts ranged from 14,806 to 95,396 for each library.

To further identify novel miRNAs in the developing Tartary buckwheat seed, the remaining sRNA sequences that were not mapped to the Tartary buckwheat reference genome were analyzed using the miRdeep2 program. As a result, a total of 129 novel miRNAs were identified (Additional file 1: Table S4). The length of these novel miRNAs ranged from 18 nt to 24 nt, and the minimum free energy (AMFE) distribution ranged from − 116.2 kcal moL^− 1^ to − 26.2 kcal moL^− 1^ (Additional file 1: Table S4). Among these novel miRNAs, 66 miRNAs were assigned to 35 known miRNA families in the miRNA database, while the remaining 63 miRNAs had no similarity to any known family. Like the above identified conserved miRNAs, more than two-thirds of the novel miRNAs were expressed at a moderate or high level, and the highest expression was fta_novel_miR71 (Additional file 1: Table S4).

### miRNAs target prediction and functional analysis

To understand the potential function of these identified miRNAs, the putative target genes of the 230 miRNAs were predicted using the TargetFinder software. A total of 3268 potential target genes were successfully predicted for among 213 miRNAs, including 2052 target genes from 101 known miRNAs and 1372 target genes from 112 novel miRNAs (Additional file 1: Table S5). These predicted target genes mainly encoded transcription factors (TFs, 285), protein kinases (254), phosphatase, E3 ubiquitin-protein ligase, proteins in hormone signal transduction and other cellular processes, and enzymes in various metabolisms (Additional file 1: Table S6). The main target TFs of these miRNAs are displayed in Additional file 1: Table S7, and the top three TFs were MYB (65), AP2/ERF (18), and NAC (18), respectively. Consistent with the results of previous studies, some conserved miRNAs targeted the known TFs. For instance, miR156, miR160, miR164, miR171, miR172, miR319, miR396, and miR858 targeted SPL, ARF, NAC, GRAS, AP2-ERF, TCP, GRF, and MYB TFs, respectively (Additional file 1: Table S7). In addition, based on a homologous search of the putative target genes, the predicted target genes of 61 miRNAs, including 39 conserved and 22 novel miRNAs, were found to be the homologous genes of 47 known seed or organ size genes (Additional file 1: Table S8). Among these, the 39 conserved miRNAs were from 14 known miRNA families including MiR156/157 (10), MiR159 (1), MiR162 (1), MiR164 (1), MiR166 (2), MiR167 (1), MiR168 (4), MiR169 (1), MiR172 (5), MiR319 (2), MiR390 (2), MiR395 (2), MiR396 (4), and MiR530 (3). Notably, among these 61 miRNAs, most miRNAs were first found to target the known seed or organ size genes, except the members of the MiR156/157 and MiR396 families which are well known to target the seed size SPL and GRF TFs, respectively. Furthermore, based on a homologous search, 8 miRNAs (4 known and 4 novel) were identified to target the structural or regulatory genes of flavonoid biosynthesis (Additional file 1: Table S9). Among these, fta_novel_miR26 targeted phenylalanine ammonia-lyase (*PAL*), fta_novel_miR74 targeted 4-coumarate-CoA ligase (*4CL*), fta-miR394a-5p targeted flavonol synthase (*FLS*), fta_novel_miR1 targeted the homologous genes of *AtMYB123* that regulate anthocyanindins biosynthesis, fta_novel_miR58 targeted the homologous genes of *AtbHLH42*/*TT8* that regulate anthocyanindins biosynthesis, and fta-miR828-5p targeted the homologous genes of *AtMYB75*/*90*/*113*/*114* that regulate anthocyanindins biosynthesis. In addition, fta-miR858a-5p and fta-miR858b-3p targeted 13 and 14 MYB TFs involved in the regulation of flavonol biosynthesis and anthocyanindins biosynthesis, respectively.

### DEMs in the development Tartary buckwheat seed and their targets

**Fig. 4:**
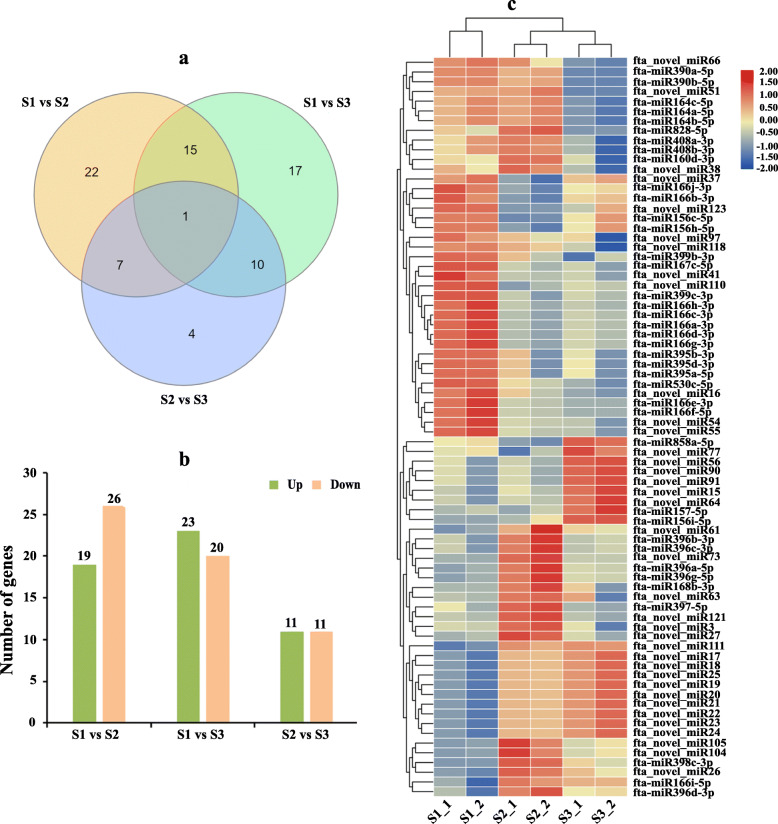
DEMs during Tartary buckwheat seed development. **a** Venn diagram represented the overlap of DEMs among the comparisons. **b** Number of DEMs in the comparisons of S1 vs. S2, S1 vs. S3, and S2 vs. S3. Green and yellow bars represented the number of up- or down-regulated genes, respectively. **c** Expression heat map of DEMs. The different colors represent the expression abundance of the miRNAs

To identify Tartary buckwheat seed development-associated miRNAs and understand their potential regulatory mechanisms, the DEMs were identified by comparing the TPM expression value. A total of 76 miRNAs, including 39 conserved and 37 novel miRNAs, displayed significant differential expression (Fig. 4). Among these, 45, 43, and 22 miRNAs were found in the comparisons between S1 and S2, S1 and S3, and S2 and S3, respectively (Fig. 4a). When compared with S1, 1 miRNA (fta_novel_miR110) was differentially expressed in both S2 and S3 stages, and 22 and 17 miRNAs were specifically differentially expressed in the S2 and S3 stage, respectively (Fig. 4a). When compared with S2, 4 miRNAs were specifically differential expression in S3 stage (Fig. 4a). In addition, between stages S1 and S2, 19 and 26 miRNAs were up- or down-regulated, respectively (Fig. 4b). In the S1 vs. S3 comparison, 23 up-regulated and 20 down-regulated miRNAs were found. In the comparison of S2 vs. S3, 11 and 11 miRNAs were up- or down-regulated, respectively (Fig. 4b).

**Fig. 5:**
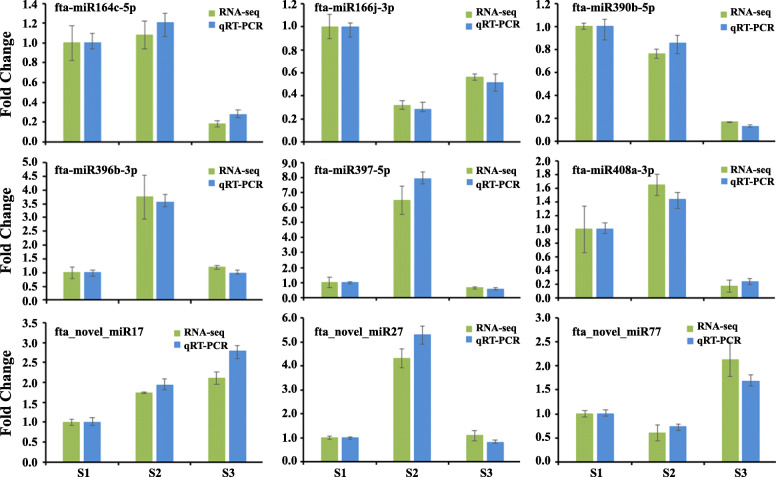
Quantitative real-time PCR verification of 9 DEMs at different developmental stages in Tartary buckwheat seeds

The expression heat map of all DEMs as shown in Fig. 4c. Among these DEMs, 26, 18, and 9 miRNAs displayed specifically high expression at the initial filling stage, peak filling stage, and initial maturity stage, respectively. Twelve miRNAs exhibited high expression at both the initial filling stage and peak filling stage. Notably, the expression of 9 novel miRNAs (fta_novel_miR17–25) was sustained growth during seed development (Fig. 4c). To verify the results of miRNA sequencing, quantitative stem-loop RT-PCR was performed to determine the expression level of 9 DEMs during Tartary buckwheat seed development. As shown in Fig. 5, the results were broadly consistent with those obtained in miRNA sequencing.

To better understand the functions of these DEMs, their predicted target genes were investigated and further subjected to Gene Ontology (GO) and Kyoto Encyclopedia of Genes and Genomes (KEGG) pathway analyses. A total of 1534 target genes were identified for among 74 DEMs (Additional file 1: Table S5). Of these target genes, 850, 765, and 735 genes were targeted by 44, 41, and 22 DEMs in the S1 vs. S2, S1 vs. S3, and S2 vs. S3 comparisons, respectively. GO analysis showed that 514, 448, and 419 targets were assigned to biological processes, cellular component, and molecular function categories for the S1 vs. S2, S1 vs. S3, and S2 vs. S3 comparisons, respectively (Additional file 2: Figure S2). There were 18, 14, and 14 GO terms in biological processes, cellular component, and molecular function categories for each comparison, respectively. The “metabolic process”, “cell part”, and “catalytic activity” were the greatest abundance terms for these three categories (Additional file 2: Figure S2). KEGG analysis displayed that a total of 332, 309, and 272 target genes were assigned to 61, 54, and 55 KEGG pathways for the S1 vs. S2, S1 vs. S3, and S2 vs. S3 comparisons (Additional file 1: Table S10), respectively. Most of these pathways were involved in the metabolism process, of which biosynthesis of “amino acids biosynthesis” (ko01230), “starch and sucrose metabolism” (ko00500) “phenylpropanoid biosynthesis” (ko00940), “purine metabolism” (ko00230), and “amino sugar and nucleotide sugar metabolism” (ko00520) were the major pathways. In addition, a high number target genes were assigned to “plant hormone signal transduction” (ko04075). The top 50 pathways for each comparison are shown in Additional file 2: Figure S3.

### Identification of the key miRNA-mRNA pairs related to Tartary buckwheat seed development

**Fig. 6:**
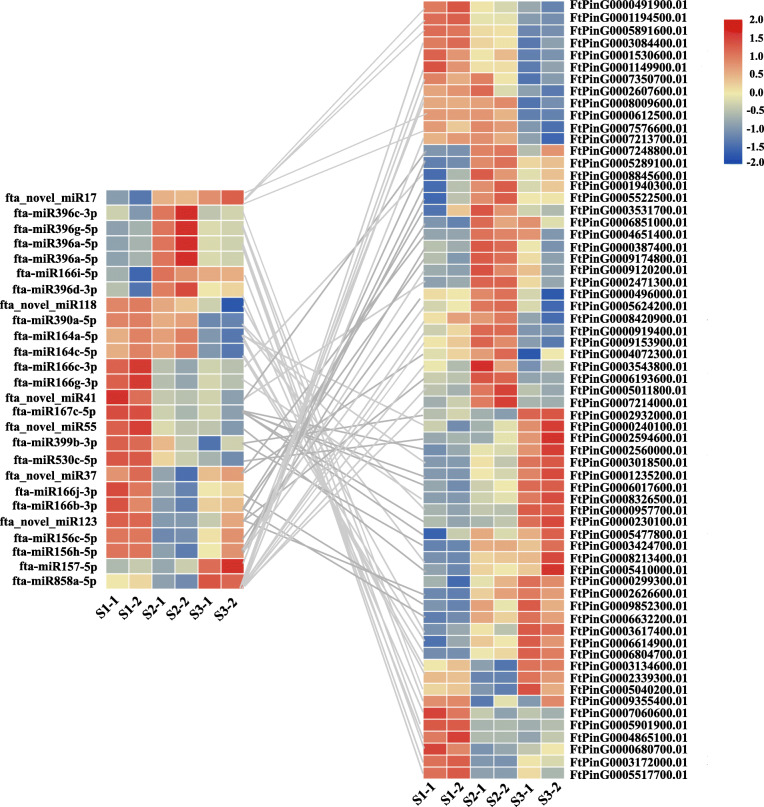
miRNA-mRNA interaction pairs showed significant negative correlation (R ≥ 0.5, *P* < 0.05) of expression during Tartary buckwheat seed development. Left: Heat map of DEMs. Right: Heat map of the differentially expressed target mRNAs of DEMs. The different colors represent the expression abundance of the miRNAs and their corresponding target mRNAs

To identify potential miRNA-mRNA pairs related to Tartary buckwheat seed development, we performed expression correlation analyses between DEMs and target mRNAs that were differentially expressed during Tartary buckwheat seed development. Based on our previous transcriptome data [46], which have the same samples with small RNA sequencing, 439 out of 1534 target genes of 74 DEMs exhibited significant differential expression during Tartary buckwheat seed development. Correlation analyses between the 439 DEGs and their corresponding DEMs identified that 117 miRNA-mRNA pairs were negatively correlated (Additional file 1: Table S11). Among them, 65 miRNA-mRNA pairs, which consisted of 25 miRNAs and 65 target genes, showed significantly expression negative correlation (R ≥ 0.5, *P* < 0.05) (Table 1 and Fig. 6). Based on a homologous annotation of these 65 target genes, 56 miRNA-mRNA pairs achieved homologous functional annotation (Table 1). These 56 miRNA-mRNA pairs were found to be significantly involved in cell differentiation and proliferation, cell elongation, hormone response and balance, organogenesis, embryo and endosperm development, transport of mineral elements (Pi, Fe and Mn), and fatty acid and flavonoid biosynthesis (Table 1). In addition, 2 calcium binding proteins, 1 receptor-like kinase, 1 CBL-interacting protein kinase, and 1 SUMO protease were identified in these 65 target genes, although their homologues in other plants were not reported to participate in seed development (Table 1). Notably, among these identified miRNA-mRNA pairs, 5 miRNA-mRNA pairs, including fta-miR156c-5p-*FtPinG0000496000.01*, fta-miR167c-5p-*FtPinG0002560000.01*, fta-miR396a-5p-*FtPinG0000680700.01*, fta-miR530c-5p-*FtPinG000259–4600.01*, and fta_novel_miR17-*FtPinG0007576600.01*, might be involved in seed size regulation of Tartary buckwheat because the target mRNAs were homologous to the known genes related to rice seed size (Table 1). Furthermore, fta-miR858a-5p exhibited conspicuous negative correlation with several MYB TFs which are the well-known regulators of plant flavonoid biosynthesis (Table 1).

**Table 1:** Identified key miRNA-mRNA interaction pairs for tartary buckwheat seed development

miRNA	Target gene ID	Annotation of targets	Function of targets	Orthologs	Pearson	***P***-value
fta-miR156c-5p	*FtPinG0000496000.01*	Squamosa promoter binding-like protein 16	Grain size and quality	*OsSPL16/Os08g0531600*	−0.70892	0.00340
fta-miR156h-5p	*FtPinG0001940300.01*	Nodulin MtN21-like transporter family protein	NA	*AT1G21890.1*	−0.86151	0.00021
	*FtPinG0005289100.01*	Ceramide synthase	Long chain fatty acid biosynthesis	*AT1G13580.1*	−0.91146	0.00033
	*FtPinG0005522500.01*	JAZ protein 9	Response to jasmonic acid	*AT1G70700.1*	−0.91696	0.00024
	*FtPinG0005624200.01*	CELLULOSE SYNTHASE-INTERACTIVE PROTEIN 1	Cell elongation	*AT2G22125.1*	−0.80006	0.00044
	*FtPinG0009120200.01*	Sulfite exporter TauE/SafE family protein	NA	*AT1G61740.1*	−0.93448	0.00228
fta-miR157-5p	*FtPinG0007350700.01*	DNA replication protein	Cell proliferation, embryo development, pollen development,	*AT2G16440.1*	−0.87897	0.00077
	*FtPinG0008009600.01*	NPH3-like protein	Involved in auxin-mediated organogenesis	*AT4G37590.1*	−0.92728	0.00073
fta-miR164a-5p	*FtPinG0000240100.01*	NAC domain-containing protein 1	Involved in shoot apical meristem formation and auxin-mediated lateral root formation, auxin response, plant growth and abiotic stress resistance	*AT1G56010.1*	−0.89303	0.00275
	*FtPinG0003134600.01*	NAC domain-containing protein 1	Involved in shoot apical meristem formation and auxin-mediated lateral root formation, auxin response, plant growth and abiotic stress resistance	*AT1G56010.1*	−0.818700	0.00281
	*FtPinG0002339300.01*	NAC domain-containing protein 5	NA	*AT5G61430.1*	−0.83097	0.00842
fta-miR164c-5p	*FtPinG0006614900.01*	Putative ligands homologous to the Clavata3 gene	Regulates stomatal and vascular development, stomatal closure	*AT1G26600.1*	−0.75278	0.00301
fta-miR166b-3p	*FtPinG0006632200.01*	Mn transporter 8	Mn transport, Mn and Fe enrichment in seed embryos	*AT3G58060.1*	−0.68351	0.00137
	*FtPinG0006851000.01*	Glutathione transferase 8	Auxin, cytokinin, drought, and oxidative stress response	*AT1G78380.1*	−0.84863	0.02431
	*FtPinG0009174800.01*	Uncharacterized protein	NA	*AT5G54980.1*	−0.82859	0.00233
fta-miR166c-3p	*FtPinG0008845600.01*	Homeodomain-leucine zipper protein 8	Involved in cell differentiation and proliferation, auxin response	*AT4G32880.1*	−0.77425	0.00131
fta-miR166g-3p	*FtPinG0008326500.01*	NA	NA	*NA*	−0.64002	0.00146
fta-miR166i-5p	*FtPinG0004865100.01*	Pentatricopeptide repeat protein	Cell proliferation during embryogenesis, embryo development	*AT3G06430.1*	−0.95992	0.000002
fta-miR166j-3p	*FtPinG0009852300.01*	Clathrin adaptor complexes medium subunit family protein	Vesicle-mediated transport	*AT1G10730.1*	−0.66005	0.00071
fta-miR167c-5p	*FtPinG0002560000.01*	NAC domain-containing protein	Grain size	*NAC024/Os05g0415400*	−0.73942	0.00003
	*FtPinG0005477800.01*	RING/U-box superfamily protein	NA	*AT1G24440.1*	−0.79803	0.00003
fta-miR390a-5p	*FtPinG0008213400.01*	Polygalacturonase inhibiting protein	Involved in defense response	*AT5G06860.1*	−0.82038	0.00261
fta-miR396a-5p	*FtPinG0000680700.01*	Sucrose synthase	Starch and cellulose biosynthesis, grain size and grain weight	*SUS3/Os07g0616800*	−0.72690	0.00059
	*FtPinG0003172000.01*	Neutral ceramidase	Sphingolipid homeostasis, oxidative stress responses, ceramide catabolic process, long-chain fatty acid biosynthetic	*AT1G07380.1*	−0.78815	0.00060
	*FtPinG0005517700.01*	Phosphoglucomutase 2	Plant growth; seed, root, male and female gametophyte development, and carbohydrate partitioning	*AT1G70730.1*	−0.69606	0.00076
fta-miR396c-3p	*FtPinG0005040200.01*	Sulfur dioxygenase	Embryo and endosperm development, abiotic stress resistance,	*T1G53580.1*	−0.778492	0.00799
fta-miR396d-3p	*FtPinG0007060600.01*	Kinase interacting (KIP1-like) family protein	NA	*AT2G22560.1*	−0.65160	0.01957
fta-miR396g-5p	*FtPinG0005901900.01*	Chloroplast-localized protein with a zinc finger motif and four GTP-binding domains	Brassinosteroid responses, positive regulation of carotenoid and chlorophyll biosynthetic process	*AT3G57180.1*	−0.59428	0.00063
	*FtPinG0009355400.01*	Chloroplast RNA editing factor	NA	*AT1G08070.1*	−0.54344	0.00062
fta-miR399b-3p	*FtPinG0002932000.01*	Alkaline/neutral invertase	Modulating hormone balance, sucrose catabolic	*AT3G06500.1*	−0.57593	0.00190
	*FtPinG0003424700.01*	Phosphate transporter	Phosphate transport and homeostasis	*AT3G54700.1*	−0.81570	0.00232
fta-miR530c-5p	*FtPinG0002594600.01*	GRF1-interacting factor 3	Regulates the sizes of stems, leaves, and grains	*OsGIF1/Os03g0733600*	−0.59617	0.01955
fta-miR858a-5p	*FtPinG0000919400.01*	MYB transcription factor 13	Regulation of flower development, responses to cold stress	*AT1G06180.1*	−0.91268	0.00001
	*FtPinG0001149900.01*	RNA recognition motif (RRM)-containing protein	Involved in phytochrome B signal transduction	*AT5G25060.1*	−0.53630	0.00001
	*FtPinG0002607600.01*	Parallel spindle 1	Pollen development	*AT1G34355.1*	−0.71400	0.00001
	*FtPinG0003084400.01*	DNA-binding bromodomain-containing protein	NA	*AT5G55040.1*	−0.54089	0.00001
	*FtPinG0003543800.01*	MYB transcription factor 7	Inhibition of seed germination	*AT2G16720.1*	−0.76017	0.00001
	*FtPinG0005011800.01*	MYB transcription factor 5	Regulation trichome and endosperm development	*AT3G13540.1*	−0.79056	0.00001
	*FtPinG0007213700.01*	MYB transcription factor 123	Regulation the proanthocyanidin accumulation of developing seed	*AT5G35550.1*	−0.89174	0.00001
	*FtPinG0007214000.01*	MYB transcription factor 23	Regulate trichome formation	*AT5G40330.1*	−0.797669	0.00001
	*FtPinG0008420900.01*	MYB transcription factor 103	Regulate cellulose and lignin biosynthesis	*AT1G63910.1*	−0.934255	0.00001
	*FtPinG0009153900.01*	MYB transcription factor 12	Regulate flavonol biosynthesis	*AT2G47460.1*	−0.935509	0.00001
fta_novel_miR17	*FtPinG0000491900.01*	receptor-like kinase	Regulation of secondary growth, protein phosphorylation	*AT5G67280.1*	−0.98523	0.00002
	*FtPinG0000612500.01*	Calcium binding protein	Involved in photomorphogensis	*AT4G08810.1*	−0.75677	0.00002
	*FtPinG0001194500.01*	Calcium binding protein	Involved in photomorphogensis	*AT4G08810.1*	−0.98204	0.00002
	*FtPinG0005891600.01*	SUMO protease	Regulation flowering, shoot and inflorescence development.	*AT4G15880.1*	−0.96316	0.00002
	*FtPinG0007576600.01*	Argonaute protein	Regulate the development of embryo and shoot meristem development, embryo development, seed size	*AGO17/AK240838*	−0.54760	0.00002
fta_novel_miR37	*FtPinG0004072300.01*	CBL-interacting protein kinase	Response to salt and osmotic stress, protein phosphorylation	*AT5G57630.1*	−0.56995	0.00126
	*FtPinG0004651400.01*	Intrinsic thylakoid membrane protein	NA	*AT2G17750.1*	−0.87363	0.00773
fta_novel_miR41	*FtPinG0002471300.01*	Carboxyesterase 13	NA	*AT3G48700.1*	−0.55115	0.00159
	*FtPinG0007248800.01*	Receptor-like protein kinase	Defense response to bacterium, protein phosphorylation	*AT4G23180.1*	−0.79183	0.00150
fta_novel_miR55	*FtPinG0005410000.01*	MYB transcription factor 73	Auxin and abiotic stress responses a	*MYB73/AT4G37260.1*	−0.63527	0.01789
fta_novel_miR118	*FtPinG0003617400.01*	GRAS transcription factor 15	Cell differentiation, repressing the seed maturation programme,	*AT4G36710.1*	−0.88771	0.01772
fta_novel_miR123	*FtPinG0000299300.01*	C2H2 zinc finger protein	Transfer cell differentiation	*AT4G27240.1*	−0.65706	0.00569
	*FtPinG0003531700.01*	CONSTANS-like 5	Flower development, and flowering	*AT5G57660.1*	−0.65534	0.00585
	*FtPinG0006913600.01*	Galactosyltransferase family protein	Embryo development	*GALT31A/AT1G32930.1*	−0.64824	0.00570

### qRT-PCR and 5′-RLM-RACE validation of the key miRNA-mRNA pairs related to Tartary buckwheat seed development

**Fig. 7:**
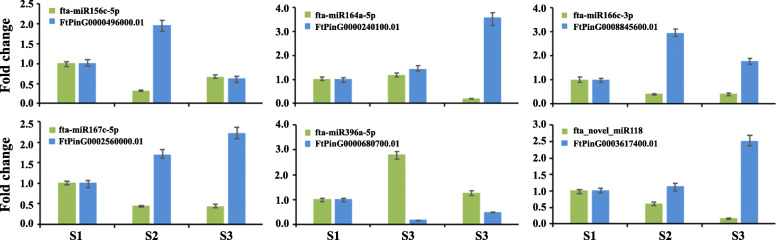
Quantitative real-time PCR validation of six identified key miRNA-mRNA interaction pairs for Tartary buckwheat seed development. Green and blue bars represented the miRNAs and their corresponding target mRNAs, respectively. The error bar represents the error values of three biological replicates

**Fig. 8:**
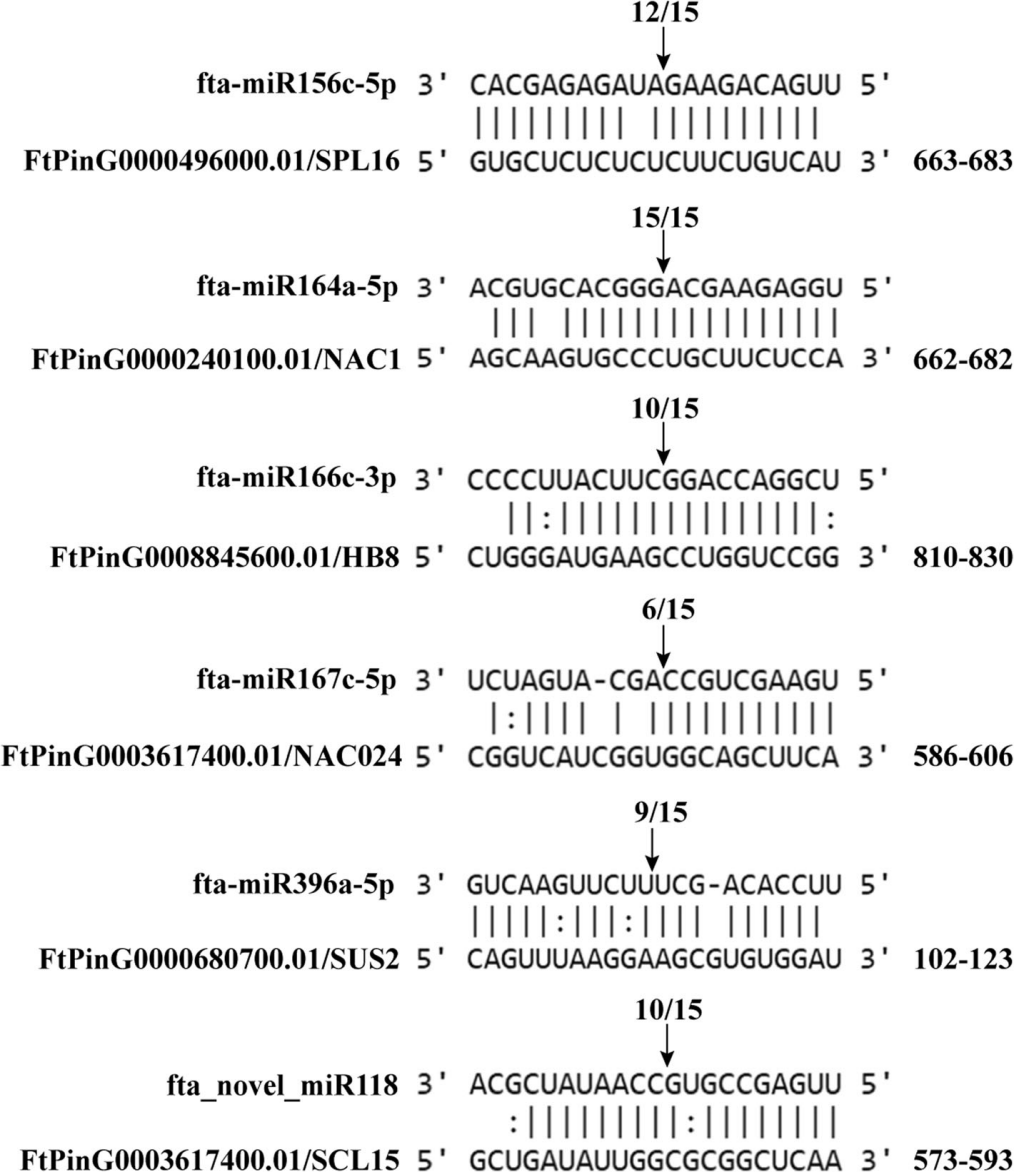
5′ RLM-RACE verification of six identified key miRNA-mRNA interaction pairs for Tartary buckwheat seed development. The numbers above sequences indicate the detected cleavage site of independent clones

To further confirm these identified key miRNA-mRNA pairs related to Tartary buckwheat seed development, qRT-PCR and 5′-RLM-RACE were performed to analyze fta-miR156c-5p-*FtPinG0000496000.01*, fta-miR164a-5p-*FtPinG0000240100.01*, fta-miR166c-3p-FtPinG0008845600.01, fta-miR167c-5p-*FtPinG0002560000.01*, fta-miR396a-5p-*FtPinG0000680700.01*, and fta_novel_miR118-*FtPinG0003617400.01* pairs, which were involved in seed size, cell differentiation and proliferation, and auxin response, respectively. qRT-PCR results indicated that the expression of these miRNAs was obviously negatively correlated with their corresponding target genes expression (Fig. 7). 5′-RLM-RACE results showed that these miRNAs could cleave their corresponding target genes (Fig. 8).

## Discussion

MiRNAs, a class negative regulators of gene expression, play key roles in numerous plant developmental processes including seed development [10, 51]. However, it whether and how to regulate the development of Tartary buckwheat seed remains unexplored. Here, to better understand the miRNAs-mediated molecular mechanisms of Tartary buckwheat seed development, we systematically identified the miRNA biosynthesis genes in the Tartary buckwheat genome and miRNA during Tartary buckwheat seed development. More importantly, we performed integrated analysis of miRNA and target mRNA expression profiles during Tartary buckwheat seed development, and identified the key miRNA-mRNA pairs for Tartary buckwheat seed development.

### miRNA biosynthesis orthologs within the Tartary buckwheat genome

Numerous studies have shown that several different genes, including *RDR*, *DCL*, *HYL*, *SE*, *HEN*, *HST*, and *AGO*, play crucial roles in plant miRNA biosynthesis [5]. In this study, we identified 8 *RDR*, 4 *DCL*, 1 *HYL*, 2 *SE*, 1 *HEN*, 2 *HST*, and 8 *AGO* genes in the Tartary buckwheat genome. The phylogenetic analyses showed that 8 RDR, 4 DCL, and 8 AGO could be divided into 4, 4, and 3 subfamilies, respectively. The results were consistent with the previous studies in other plants [6, 49, 50, 52, 53], and indicated that the evolution of these gene families was conserved in these plants. Expression analysis revealed that 1 *AGO*-like *FtAGO1* had the highest expression in seeds in these identified genes, 1 *AGO*-like *FtAGO4* exhibited specific high expression in seeds, and 2 *AGO*-like *FtAGO2* and *FtAGO3* exhibited significant differential expression during seed development. Notably, *OsAGO17*, which belongs to the same subfamily as these four *FtAGO* genes, had been functionally demonstrated to positively regulate the grain size and grain weight in rice [54, 55]. This suggested that these four *FtAGO* genes might also play a similar role with *OsAGO17* in Tartary buckwheat seed development. In addition, we also found that *FtHYL1*, *FtHST2*, and *FtAGO8* displayed especially high expression in seeds, and *FtDCL1*, *FtDCL3*, *FtDCL4*, and *FtRDR4* were obviously differential expression during seed development. These indicated that these miRNA biosynthesis genes might also have important regulatory roles in Tartary buckwheat seed development.

### Characteristics of sRNAs during Tartary buckwheat seed development

By sequencing six sRNA libraries from three differently developmental stages seeds, we obtained abundant sRNAs with a length of 18–30 nt. Among them, the 24 nt sRNAs were the most abundant in developmental Tartary buckwheat seed, which was similar to previous observations in the developmental seed of many plants [6, 56]. Furthermore, the 24 nt sRNAs exhibited differential accumulation during Tartary buckwheat seed development. These suggested that the 24 nt sRNAs might play crucial roles in Tartary buckwheat and other plant seed development. It has been reported that many of the 24-nt sRNAs were heterochromatic siRNAs (hetsiRNAs), which mediate transcriptional gene silencing through DNA methylation (RdDM) [56]. Generally, the 24-nt sRNAs need from cytoplasm imported into the nucleus before they were methylated, and the process was mediated by AGO4 [57]. Notably, our study observed that *FtAGO8*, which was the *AGO4* ortholog, exhibited special high expression in the development Tartary buckwheat seed. These observations implied that *FtAGO8* might has a role similar to *AGO4* in 24-nt sRNAs methylation, and these 24-nt sRNAs might mediate transcriptional gene silencing through the RdDM pathway during Tartary buckwheat seed development.

### Known and novel miRNAs and their target genes in the developmental Tartary buckwheat seed

In the Tartary buckwheat genome, 278 miRNAs have been predicted through a genome-wide bioinformatics analysis [58]. In our study, a total of 230 miRNAs, including 101 known and 129 novel miRNAs, were identified during Tartary buckwheat seed development. Among the miRNAs, more than two-thirds were expressed at high and moderate levels. These indicated that most miRNAs in the Tartary buckwheat genome were involved in the seed development, and further suggested that Tartary buckwheat seed development was an extremely complex biological process. Notably, the fta-miR159 exhibited the highest expression in the developmental Tartary buckwheat seed. In strawberry, Fa-miR159b and Fa-miR159b were more highly expressed in developing fruit and played a key regulatory role in fruit development [4, 59]. In rice, osa-miR159 was also more highly expressed in developing seed and positively regulated grain length and width [4, 20, 33, 35]. Therefore, our results suggested that fta-miR159 might also possess a crucial regulation role in the Tartary buckwheat seed development.

Using TargetFinder software, we identified 3268 potential target genes from 213 miRNAs. Among these target genes, the largest number were encoded TFs. Notably, among these miRNAs with target gene encoded TFs, some miRNAs such as miR156, miR160, miR396, and miR858 targeted the SBP, ARF, GRF, and MYB TFs, respectively. Interestingly, these miRNAs have been experimentally verified to target these corresponding TFs in other plants [32, 36, 37, 60]. This suggested that these miRNA targets were highly conserved in different plants and further confirmed the high reliability of target identification in our study. Based on a homologous search of the predicted target genes, 61 miRNAs have target genes were homologous genes of 47 known seed or organ size genes [61]. Notably, among these miRNAs, most were first identified targeting the known seed or organ size genes, except for miR156 and miR396 targeting the known seed size SPL and GRF TFs, respectively [22, 32, 36]. These showed that these miRNAs might play an important regulatory role in Tartary buckwheat seed size and most of these known seed or organ size regulatory genes might exist a post-transcription regulatory mechanism in regulating seed size. In plants, miRNAs have been reported to regulate flavonoid biosynthesis through target regulating the structural or regulatory genes of flavonoid biosynthesis [62]. In our study, 3 miRNAs (fta_novel_miR26, fta_novel_miR74, and fta-miR394a-5p) were identified to target the flavonoid biosynthesis structural genes *PAL*, *4CL*, and *FLS*, respectively. Furthermore, 5 miRNAs (fta_novel_miR1, fta_novel_miR58, fta-miR858a-5p, fta-miR858a-5p, and fta-miR858b-3p) were found to target the orthologs of known flavonoid biosynthesis regulatory genes in *Arabidopsis thaliana* [63–66]. These implied that these miRNAs could regulate flavonoid biosynthesis in Tartary buckwheat by regulating the expression of the structural or regulatory genes of flavonoid biosynthesis.

### DEMs during Tartary buckwheat seed development

In many plants, a larger number of DEMs have been identified in the developing seed [4, 6, 18–30]. In this study, we identified 76 DEMs during Tartary buckwheat seed development. Expression profiles analysis revealed that these DEMs possessed stage-specific highly expressed patterns during Tartary buckwheat seed development, suggesting that these DEMs might perform their regulatory roles in a specific stage during Tartary buckwheat seed development and miRNAs with the same expression pattern might have a similar regulatory role in the developing Tartary buckwheat seed. Notably, some DEMs such as miR156, miR160, miR166, miR167, miR168, miR395, miR396, miR397, miR398, miR399, and miR408 were also found to be differentially expressed during seed development in many seed plants [4, 18, 19, 21–30]. In rice, miR156, miR160, miR167, miR396, miR397, miR389, and miR408 have been functionally verified to regulate the rice seed development [31–38, 40]. These suggested that these known miRNAs were the key and conserved miRNAs in different plant seed development regulation and could also be used as candidate miRNAs for Tartary buckwheat seed development. In addition, 37 novel miRNAs and several conserved miRNAs, including miR530, miR828, and miR858, were found to be specifically differential expression in developing Tartary buckwheat seed when compared with other seed plants. This indicated that these miRNAs might have a specific regulatory function in Tartary buckwheat seed development. Furthermore, all these information indicated that conserved and diverse miRNA-mediated regulatory mechanisms in seed development might exist in different seed plants.

### Integration analysis of miRNA and target mRNA

Integrated miRNA and its target mRNA expression analysis could be helpful in understanding the function of miRNAs and identifying the functional miRNA-mRNA pairs related to seed development [67]. To our knowledge, there was no integration analysis of miRNA and target mRNA performed in previous studies of plant seed development. In this study, we identified 65 significantly negatively correlated miRNA-mRNA pairs (*R* ≥ 0.5, *P* < 0.05), which consisted of 25 miRNAs and 65 corresponding target genes. Based on homologous queries of these miRNAs corresponding target genes, 56 miRNA-mRNA pairs obtained functional annotations. These miRNA-mRNA pairs are involved in different aspects, most significantly cell differentiation and proliferation, cell elongation, hormones response and balance, organogenesis, embryo and endosperm development, mineral elements transport and accumulation, and flavonoid biosynthesis. These suggested that these identified miRNA-mRNA pairs were the key miRNA-mRNA pairs related to Tartary buckwheat seed development.

In rice, *OsSPL16* [68, 69], *OsNAC024* [70], *OsSUS* [71], *OsGIF1* [38, 72], and *OsAGO17* [54] genes have been functionally demonstrated to regulate the seed size. In this study, we found the target mRNAs from 5 miRNA-mRNA pairs (fta-miR156c-5p-FtPinG0000496000.01, fta-miR167c-5p-FtPinG0002560000.01, fta-miR396a-5p-FtPinG0000680700.01, fta-miR530c-5p-FtPinG0002594600.01 and fta_novel_miR17-FtPinG0007576600.01) were homologous with these 5 rice seed size genes, respectively, which indicated that these genes might have a conserved regulatory role in the seed size of Tartary buckwheat seed and the corresponding miRNAs were the key miRNAs for controlling Tartary buckwheat seed size. Notably, to date, these seed size genes had not been identified as the target genes of any miRNAs in previous studies. Therefore, our results provided the first-hand information on the post-transcription regulation of these seed size genes. It is well known that the Ca^2+^ signal transduction pathways play important roles in many developmental processes including seed development in plants [73, 74]. In this study, we found that three of the 56 identified miRNA-mRNA pairs were involved in Ca^2+^ signal transduction because the target mRNAs were homologous to the calcium binding protein and CBL-interacting protein kinases, respectively. This suggested that these miRNAs could regulate Tartary buckwheat seed development though Ca^2+^ signal transduction pathways. In a previous study, we found that the total seed flavonoid content was dynamically accumulated during Tartary buckwheat seed development, and identified one SG7 subgroup R2R3-MYB TF gene (*FtPinG0009153900.01*) was the key regulatory gene of flavonoid biosynthesis in the developing Tartary buckwheat seed [46]. Interestingly, in this study, we found fta-miR858a-5p-*FtPinG0009153900.01* pair displayed significantly expression negative correlation. In addition, fta-miR858a-5p also exhibited conspicuous negative correlation with the orthologs of *Arabidopsis thaliana MYB123*, which was the well-known regulator of proanthocyanidin accumulation in developing seed [64]. These indicated that the flavonoid biosynthesis of Tartary buckwheat seed is involved in miRNA-mediated post-transcription regulation and fta-miR858a-5p is the key regulator.

To further verify the reliability of these identified miRNA-mRNA pairs related to Tartary buckwheat seed development, we carried out qRT-PCR and 5′-RLM-RACE analyses for among 6 miRNA-mRNA pairs that are involved in seed size, cell differentiation and proliferation, and auxin response. These results showed that the expression of these miRNAs and their corresponding target genes were obviously negatively related and could cleave their corresponding target genes. The above results indicated that these identified miRNA-mRNA pairs could be considered as the candidate miRNA-mRNA pairs for regulating the development of Tartary buckwheat seed, and also suggested that combining small RNA and transcriptome analysis is an effective method for identifying key miRNAs that are involved in plant seed development.

## Conclusion

The present work is the first attempt to integrate miRNA and mRNA expression data to identify key regulatory miRNA-mRNA pairs in developing Tartary buckwheat seed. A total of 230 miRNAs, including 101 known and 129 novel miRNAs, were first identified in Tartary buckwheat. Among these miRNAs, 76 showed significant differential expression during Tartary buckwheat seed development, and 1543 of their target genes were identified. Additionally, 25 miRNAs corresponding to 56 target genes were identified as key candidate miRNA-mRNA pairs for Tartary buckwheat seed development. The integrated analyses of miRNAs and target mRNAs in this study not only provide the first new insights into miRNA-mediated regulation in Tartary buckwheat seed development, but also provide a basis for further research into the functions of these candidate miRNAs and their targets in Tartary buckwheat seed development and for Tartary buckwheat seed improvement.

## Methods

### Plant materials and sample preparation

Tartary buckwheat cultivar Jinqiao No. 2, which was obtained from the Research Center of Buckwheat Industry Technology of Guizhou Normal University (Guiyang, Guizhou, China), was used in this study. The seed samples used for sRNA sequencing were same as our previous transcriptome analysis [46]. In brief, seeds were collected at 12 (S1: initial filling stage), 15 (S2: peak filling stage), and 21 (S3: initial maturity stage) days after pollination (DAP) [46].

### Identification, phylogeny, and expression profiles analysis of the miRNA biosynthesis genes in the Tartary buckwheat genome

The Tartary buckwheat genome information was downloaded from http://www.mbkbase.org/Pinku1 [58]. All protein sequences from annotated genes were used to build a local protein blast database in BioEdit software (Version 7.0.9.0). The protein sequences of *RDR1*–*6*, *DCL1*–*4*, *AGO1*–*10*, *HEN1*, *HYL1*, *SE*, and *HST* genes from *Arabidopsis thaliana* were used as queries to carry out local blastp with e-values <e^− 5^ to identify orthologs in the Tartary buckwheat genome. The conserved domain of all obtained non-redundant sequences were further examined in the Pfam (http://pfam.sanger.ac.uk) [75], and the final orthologs were ensured. The RDR, DCL, and AGO protein sequences from Tartary buckwheat, *Arabidopsis thaliana*, rice, and maize were used to construct the phylogenetic trees using the Maximum Likelihood method in MEGA 7.0.21 software. Expression analysis of these identified miRNA biosynthesis genes in Tartary buckwheat was performed using RNA-seq data from 4 organs (root, stem, leaf, and flower) [58] and three developmental-stage seeds [46]. The expression heatmaps of these genes were generated by using TBtools software [76].

### RNA extraction, sRNA library construction, and sequencing

Total RNA was extracted from three samples as previously described [46] and used for sRNA and qRT-PCR assay. RNA quality and concentration were determined via 1.2% agarose gel electrophoresis and NanoDrop 2000c spectrophotometer (NanoDrop, Wilmington, DE, USA), respectively.

For construction and sequencing of the sRNA libraries, two replicates were performed for each sample. Firstly, sRNAs were isolated from total RNA by polyacrylamide gel electrophoresis (PAGE). Next, the isolated sRNAs were added to a 5′ RNA adaptor and a 3′ RNA adaptor by using T4 RNA ligase (TaKaRa, Dalian, China). Then, sRNAs with added 5′ and 3′ RNA adaptors were reverse transcribed into single-stranded cDNA using RT-PCR. Follows, the single-stranded cDNA was further synthesized into double-stranded cDNA by PCR amplification using adapter primers. Finally, the PCR product was purified and subjected to high-throughput sequencing by using the Illumina SE50 system at Biomarker Technologies Co., Ltd. (Beijing, China).

### Bioinformatics analysis of sequencing data

Raw reads were processed to generate clean reads by removing the low-quality reads, reads containing poly-N or poly-A sequences, reads lacking the 3′ adaptor sequence, reads with length < 18 nt or > 30 nt, and adapter sequences. The clean reads were then mapped against the Tartary buckwheat genome (http://www.mbkbase.org/Pinku1/) [58]. The Silva database, GtRNAdb database, Rfam database and Repbase database were used to filter the rRNA, tRNA, snRNA, and snoRNA to produce the unannotated reads containing miRNA [4]. Unannotated reads were blasted against miRBase to search for known miRNAs [77]. The potential novel miRNAs were predicted by using the miRdeep2 program [78]. The miFam.dat (http://www.miRbase.org/ftp.shtml) was used to investigate the miRNA family class of these identified miRNAs. The miRNA expression abundances were calculated and normalized by transcript per million (TPM), and differential expression analysis was carried out by the DEGSeq R package with FDR (false discovery rate) value < 0.05 and |log2(fold change)| > 1 as the threshold for significant difference. The potential target genes of miRNA were predicted by using TargetFinder software [79]. The predicted target genes were subjected to GO (http://geneontology.org/) [80] and KEGG (www.kegg.jp/kegg/kegg1. html) [81] pathway analysis to predict and classify possible functions.

### Identification of the key miRNA-mRNA pairs for Tartary buckwheat seed development

Expression profiles of all the DEMs target genes were analyzed by using transcriptomic data from our previous report [46]. Then, the significantly different expression target genes were identified and functional analysis was performed by GO, KEGG and homologous annotation. Finally, expression correlation analysis between DEMs and differently expressed target genes was performed. The miRNAs and target mRNA pairs with *R* ≥ 0.5 and *P* value < 0.05 were identified, and the key miRNA-mRNA pairs for Tartary buckwheat seed development were obtained by homologous annotation of target mRNAs.

### qRT-PCR validation of DEMs and its differently expressed target genes

Total RNA was isolated from three different developmental stages of Tartary buckwheat seeds by using the EASYspin Plus Plant RNA Kit (Aidlab, Beijing, China) and digested by DNase I (TaKaRa, Dalian, China) to remove the genomic DNA. Then, 2 μg total RNA was reverse transcribed into single-stranded cDNA using the miRcute miRNA First-Strand cDNA Synthesis Kit (Tiangen, Beijing, China) following the manufacturer’s instructions. The qRT-PCR was performed on a CFX96 Real-time System (BIO-RAD, USA) using the miRcute Plus miRNA qPCR Kit (SYBR Green) (Tiangen, Beijing, China). Three independent biological replicates were used, and the *FtU6* was used as the reference gene. The relative expression level of each miRNA was calculated by using the 2^−ΔΔCt^ method. All primers used in this experiment are listed in Additional file 1: Table S12.

For differently expressed target genes, reverse transcription and qRT-PCR reactions were carried out by using PrimeScript™ RT Master Mix (Perfect Real Time) (TaKaRa, Beijing, China) and TB Green® Premix Ex Taq™ (Tli RNaseH Plus) (TaKaRa, Beijing, China), respectively. *FtActin* was used as the reference gene. All samples were performed three independent biological replicates. The relative expression level of each gene was calculated by using the 2^−ΔΔCt^ method. The primers used in this experiment are listed in Additional file 1: Table S12.

### Validation of the miRNA-directed cleavage of their predicted targets

The 5′-RLM-RACE method was used to investigate the miRNA-directed cleavage of their predicted target mRNA in vitro. Total RNA was ligated to an RNA adaptor and was reverse transcribed using the FirstChoice® RLM-RACE Kit (Ambion, USA) according to the manufacturer’s instructions. The 5′-RLM-RACE was performed as described previously by DeBoer et al. [6]. The primers used in this experiment are listed in Additional file 1: Table S13.

## Supplementary Information

**Additional file 1: Table S1.** Characteristic features of all the identified *DCL*, *AGO*, *HYL1*, *SE*, *HST*, and *RDRs* in tartary buckwheat genome. **Table S2.** Length distribution of sRNA sequences identified in developing tartary buckwheat seeds. **Table S3.** Information of identified conserved miRNAs. **Table S4.** Information of identified novel miRNAs. **Table S5.** Predicted target genes of miRNAs in tartary buckwheat. **Table S6.** Annotation of miRNAs target genes in tartary buckwheat. **Table S7.** Information of TFs targeting by miRNAs. **Table S8.** miRNAs have target genes are the orthologs of the known seed or organ size. **Table S9.** miRNAs have target genes are the orthologs of the known structural or regulatory genes of flavonoid biosynthesis. **Table S10.** KEGG pathways of the target genes of DEMs. **Table S11.** miRNA-mRNA interaction pairs show expression negative correlation during tartary buckwheat seed development. **Table S12.** Primers of sequences for qRT-PCR analysis. **Table S13.** Primers of sequences for RLM-5′RACE analysis.

**Additional file 2: Figure S1.** Read length distribution of sRNAs. **Figure S2.** GO analysis of the target genes of DEMs. **Figure S3.** KEGG analysis of the target genes of DEMs.

## Data Availability

The datasets supporting the conclusions of this article are included within the article and its additional files. The dataset and materials presented in the investigation is available by request from the corresponding author.

## Abbreviations

DEMs: Differentially expressed miRNAs
qRT-PCR: Quantitative real-time polymerase chain reaction
5′-RLM-RACE: Ligase-mediated rapid amplification of 5′ cDNA ends
miRNAs: MicroRNAs
sRNAs: Small RNAs
DCL1: Dicer-Like 1
HYL1: Hyponastic Leaves 1
SE: Serrate
HEN1: Hua enhancer 1
AGO1: Argonaute 1
RISC: RNA-induced silencing complex
DEGs: Differentially expressed genes
TFs: Transcription factors
hetsiRNAs: Heterochromatic siRNAs
AMFE: Minimum free energy
PAL: Phenylalanine ammonia-lyase
4CL: 4-coumarate-CoA ligase
FLS: Flavonol synthase
GO: Gene ontology
KEGG: Kyoto Encyclopedia of Genes and Genomes
DAP: Days after pollination
