# Supplementary material for: Integrated microRNA and transcriptome profiling reveal key miRNA-mRNA interaction pairs associated with seed development in Tartary buckwheat (Fagopyrum tataricum)
Source: BMC Plant Biol. 2021 Mar 9;21:132. doi: 10.1186/s12870-021-02914-w (PMC7941931; doi:10.1186/s12870-021-02914-w)
Supplement: Supplementary file 1 — Additional file 1: Table S1. Characteristic features of all the identified DCL, AGO, HYL1, SE, HST, and RDRs in tartary buckwheat genome. Table S2. Length distribution of sRNA sequences identified in developing tartary buckwheat seeds. Table S3. Information of identified conserved miRNAs. Table S4. Information of identified novel miRNAs. Table S5. Predicted target genes of miRNAs in tartary buckwheat. Table S6. Annotation of miRNAs target genes in tartary buckwheat. Table S7. Information of TFs targeting by miRNAs. Table S8. miRNAs have target genes are the orthologs of the known seed or organ size. Table S9. miRNAs have target genes are the orthologs of the known structural or regulatory genes of flavonoid biosynthesis. Table S10. KEGG pathways of the target genes of DEMs. Table S11. miRNA-mRNA interaction pairs show expression negative correlation during tartary buckwheat seed development. Table S12. Primers of sequences for qRT-PCR analysis. Table S13. Primers of sequences for RLM-5′RACE analysis. [file 12870_2021_2914_MOESM1_ESM.zip › Table S9.docx]

**Table S9. miRNAs have target genes are the orthologs of the known structural or regulatory genes of flavonoid biosynthesis**

| **miRNA** | **Target gene** | **Target annotation** | **Target functions** | **Arabidopsis homologous** |
| --- | --- | --- | --- | --- |
| fta_novel_miR26 | FtPinG0008237100.01 | Phenylalanine ammonia-lyase | Phenylpropanoid pathway | *AtPAL* |
| fta_novel_miR74 | FtPinG0003957300.01 | 4-coumarate-CoA ligase 1 | Phenylpropanoid pathway | *At4CL* |
| fta-miR394a-5p | FtPinG0008448800.01 | Flavonol synthase | Phenylpropanoid pathway / flavonol biosynthesis | *AtFLS1* |
| fta_novel_miR1 | FtPinG0003544400.01 | MYB transcription factor | Phenylpropanoid pathway / anthocyanindins biosynthesis | *AtMYB123* |
| fta_novel_miR58 | FtPinG0008212200.01 | bHLH transcription factor | Phenylpropanoid pathway / anthocyanindins biosynthesis | *bHLH42/TT8* |
| fta-miR828-5p | FtPinG0008340900.01 | MYB transcription factor | Phenylpropanoid pathway / anthocyanin biosynthesis | *AtMYB75/90/113/114* |
| fta-miR858a-5p | FtPinG0004608100.01 | MYB transcription factor | Phenylpropanoid pathway / flavonol biosynthesis | *AtMYB11/12/111* |
|  | FtPinG0009153900.01 | MYB transcription factor | Phenylpropanoid pathway / flavonol biosynthesis | *AtMYB11/12/111* |
|  | FtPinG0004494100.01 | MYB transcription factor | Negative regulation, flavonol  biosynthesis | *AtMYB7/32* |
|  | FtPinG0000631000.01 | MYB transcription factor | Phenylpropanoid pathway / anthocyanin biosynthesis | *AtMYB75/90/113/114* |
|  | FtPinG0007214000.01 | MYB transcription factor | Phenylpropanoid pathway / anthocyanin biosynthesis | *AtMYB75/90/113/114* |
|  | FtPinG0000518100.01 | MYB transcription factor | Phenylpropanoid pathway / anthocyanindins biosynthesis | *AtMYB123* |
|  | FtPinG0003041600.01 | MYB transcription factor | Phenylpropanoid pathway / anthocyanindins biosynthesis | *AtMYB123* |
|  | FtPinG0003543800.01 | MYB transcription factor | Phenylpropanoid pathway / anthocyanindins biosynthesis | *AtMYB123* |
|  | FtPinG0003544400.01 | MYB transcription factor | Phenylpropanoid pathway / anthocyanindins biosynthesis | *AtMYB123* |
|  | FtPinG0005011800.01 | MYB transcription factor | Phenylpropanoid pathway / anthocyanindins biosynthesis | *AtMYB123* |
|  | FtPinG0007213700.01 | MYB transcription factor | Phenylpropanoid pathway / anthocyanindins biosynthesis | *AtMYB123* |
|  | FtPinG0007711300.01 | MYB transcription factor | Phenylpropanoid pathway / anthocyanindins biosynthesis | *AtMYB123* |
|  | FtPinG0008341600.01 | MYB transcription factor | Phenylpropanoid pathway / anthocyanindins biosynthesis | *AtMYB123* |
| fta-miR858b-3p | FtPinG0004608100.01 | MYB transcription factor | Phenylpropanoid pathway / flavonol biosynthesis | *AtMYB11/12/111* |
|  | FtPinG0009153900.01 | MYB transcription factor | Phenylpropanoid pathway / flavonol biosynthesis | *AtMYB11/12/111* |
|  | FtPinG0004494100.01 | MYB transcription factor | Negative regulation, flavonol  biosynthesis | *AtMYB7/32* |
|  | FtPinG0000631000.01 | MYB transcription factor | Phenylpropanoid pathway / anthocyanin biosynthesis | *AtMYB75/90/113/114* |
|  | FtPinG0007214000.01 | MYB transcription factor | Phenylpropanoid pathway / anthocyanin biosynthesis | *AtMYB75/90/113/114* |
|  | FtPinG0008340900.01 | MYB transcription factor | Phenylpropanoid pathway / anthocyanin biosynthesis | *AtMYB75/90/113/114* |
|  | FtPinG0000518100.01 | MYB transcription factor | Phenylpropanoid pathway / anthocyanindins biosynthesis | *AtMYB123* |
|  | FtPinG0003041600.01 | MYB transcription factor | Phenylpropanoid pathway / anthocyanindins biosynthesis | *AtMYB123* |
|  | FtPinG0003543800.01 | MYB transcription factor | Phenylpropanoid pathway / anthocyanindins biosynthesis | *AtMYB123* |
|  | FtPinG0003544400.01 | MYB transcription factor | Phenylpropanoid pathway / anthocyanindins biosynthesis | *AtMYB123* |
|  | FtPinG0005011800.01 | MYB transcription factor | Phenylpropanoid pathway / anthocyanindins biosynthesis | *AtMYB123* |
|  | FtPinG0007213700.01 | MYB transcription factor | Phenylpropanoid pathway / anthocyanindins biosynthesis | *AtMYB123* |
|  | FtPinG0007711300.01 | MYB transcription factor | Phenylpropanoid pathway / anthocyanindins biosynthesis | *AtMYB123* |
|  | FtPinG0008341600.01 | MYB transcription factor | Phenylpropanoid pathway / anthocyanindins biosynthesis | *AtMYB123* |
