# Supplementary figures and images for: Integrated microRNA and transcriptome profiling reveal key miRNA-mRNA interaction pairs associated with seed development in Tartary buckwheat (Fagopyrum tataricum)
Source: BMC Plant Biol. 2021 Mar 9;21:132. doi: 10.1186/s12870-021-02914-w (PMC7941931; doi:10.1186/s12870-021-02914-w)

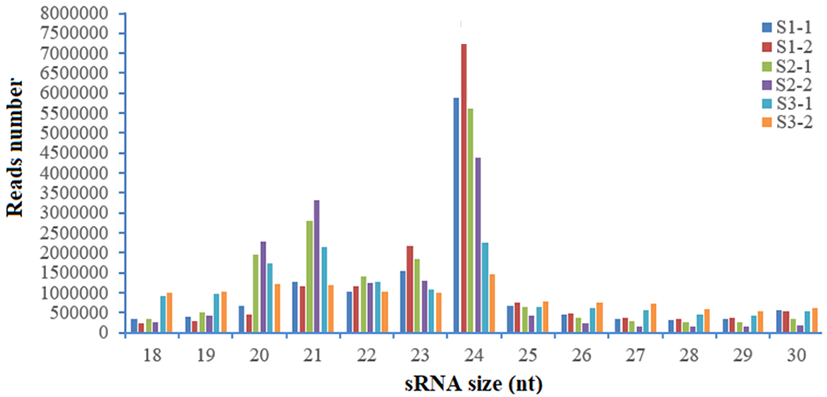

Supplement: Supplementary file 2 — Additional file 2: Figure S1. Read length distribution of sRNAs. Figure S2. GO analysis of the target genes of DEMs. Figure S3. KEGG analysis of the target genes of DEMs. [file 12870_2021_2914_MOESM2_ESM.zip › Figure S1.tif]

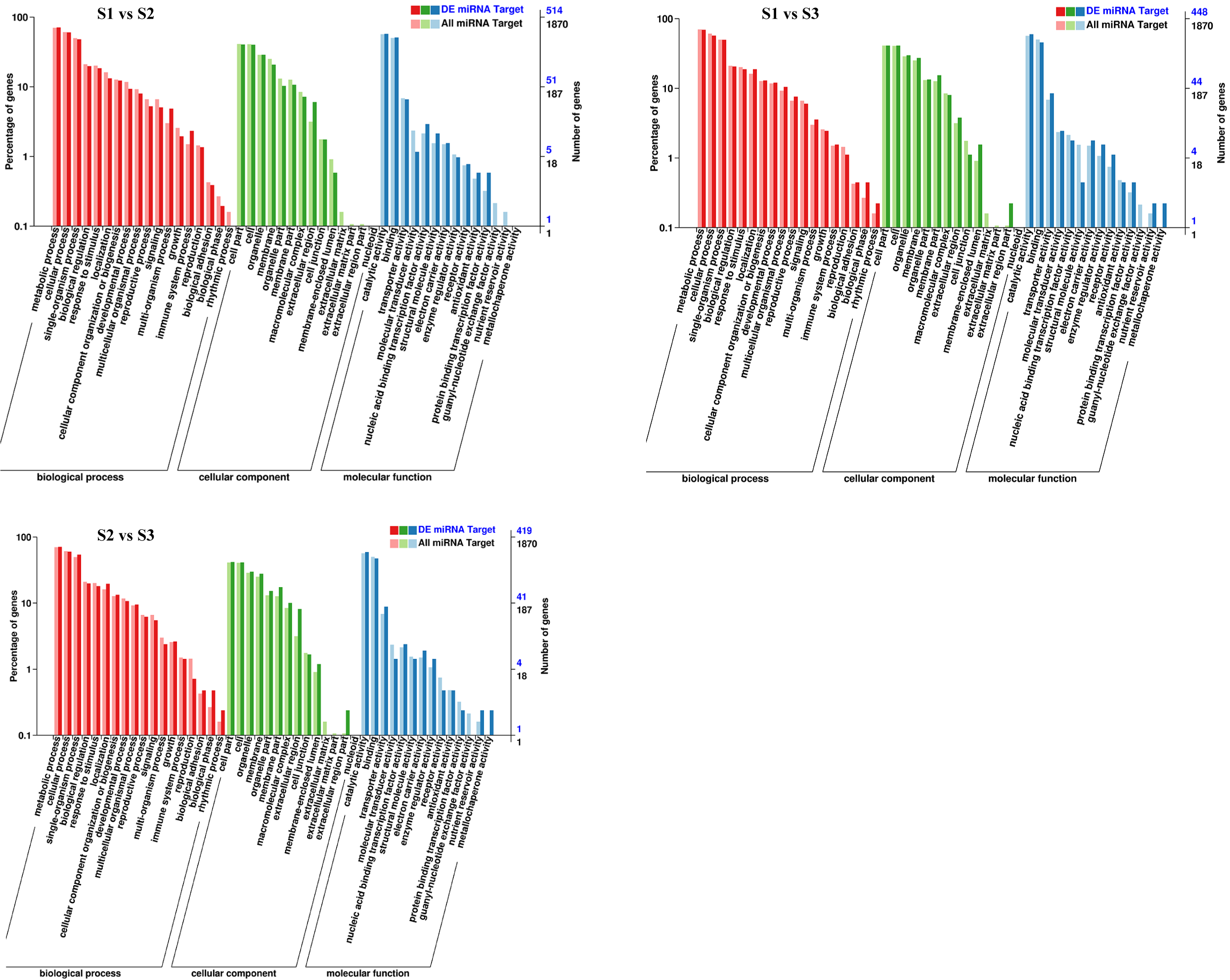

Supplement: Supplementary file 2 — Additional file 2: Figure S1. Read length distribution of sRNAs. Figure S2. GO analysis of the target genes of DEMs. Figure S3. KEGG analysis of the target genes of DEMs. [file 12870_2021_2914_MOESM2_ESM.zip › Figure. S2.tif]

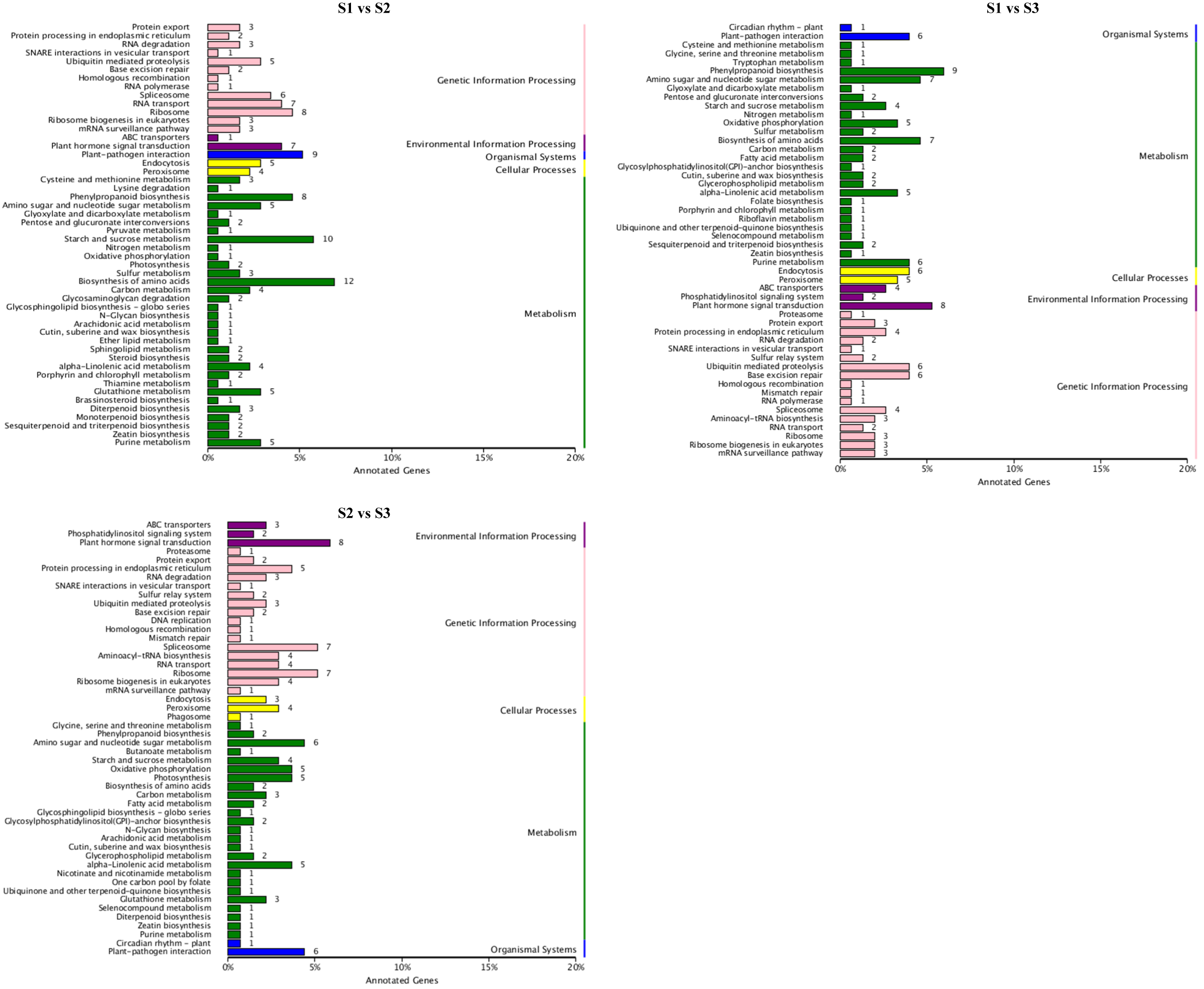

Supplement: Supplementary file 2 — Additional file 2: Figure S1. Read length distribution of sRNAs. Figure S2. GO analysis of the target genes of DEMs. Figure S3. KEGG analysis of the target genes of DEMs. [file 12870_2021_2914_MOESM2_ESM.zip › Figure. S3.tif]
